# Polyphenol Release from Wheat Bran Using Ethanol-Based Organosolv Treatment and Acid/Alkaline Catalysis: Process Modeling Based on Severity and Response Surface Optimization

**DOI:** 10.3390/antiox11122457

**Published:** 2022-12-14

**Authors:** Eirini S. Papadaki, Dimitrios Palaiogiannis, Stavros I. Lalas, Paraskevi Mitlianga, Dimitris P. Makris

**Affiliations:** 1 Laboratory of Food Chemistry and Technology, Department of Chemical Engineering, University of Western Macedonia, ZEP Campus, 50100 Kozani, Greece; 2 Green Processes & Biorefinery Group, Department of Food Science & Nutrition, School of Agricultural Sciences, University of Thessaly, N. Temponera Street, 43100 Karditsa, Greece

**Keywords:** antioxidants, ferulic acid, organosolv treatment, polyphenols, severity factor, wheat bran

## Abstract

Wheat bran (WB) is globally a major food industry waste, with a high prospect as a bioresource in the production of precious polyphenolic phytochemicals. In this framework, the current investigation had as objectives (i) to use ethanol organosolv treatment and study the effect of acid and alkali catalysts on releasing bound polyphenols, (ii) establish linear and quadratic models of polyphenol recovery based on severity and response surface, and (iii) examine the polyphenolic composition of the extracts generated. Using sulfuric acid and sodium hydroxide as the acid and the alkali catalyst, respectively, it was found that the correlation of combined severity factor with total polyphenol yield was significant in the acid catalysis, but a highly significant correlation in the alkali-catalyzed process was established with modified severity factor, which takes into consideration catalyst concentration, instead of pH. Optimization of the process with response surface confirmed that polyphenol release from WB was linked to treatment time, but also catalyst concentration. Under optimized conditions, the acid- and alkali-catalyzed processes afforded total polyphenol yields of 10.93 ± 0.62 and 19.76 ± 0.76 mg ferulic acid equivalents g^−1^ dry mass, respectively. Examination of the polyphenolic composition revealed that the alkali-catalyzed process had a striking effect on releasing ferulic acid, but the acid catalysis was insufficient in this regard. The outcome concerning the antioxidant properties was contradictory with respect to the antiradical activity and ferric-reducing power of the extracts, a fact most probably attributed to extract constituents other than ferulic acid. The process modeling proposed herein may be valuable in assessing both process effectiveness and severity, with a perspective of establishing WB treatments that would provide maximum polyphenol recovery with minimum harshness and cost.

## 1. Introduction

The current natural resources are constantly overexploited, to provide food supply to the growing world population, and at the same time, there is an ever-increasing rate of waste generation. This waste biomass is mainly dumped into sanitary landfills, resulting in the limitation of the land destined for agriculture, and causing serious damage to ecosystems. As opposed to such a linear model of development, circular economy strategies are oriented towards waste valorization, aiming at reducing the indiscriminate use of resources. Thus, food production is channeled into sustainable systems of resource restoration and regeneration, limiting the irrational use of raw materials and energy, and minimizing waste generation [1]. The waste material derived from the agri-food sector embraces residues from crop cultivation, post-harvest rejected tissues, and food processing side-streams, all of which are bio-materials rich in substances that may be used to produce fuels, energy, platform chemicals, and high value-added compounds [2]. 

Cereals represent a large part of the food industry sector, with rice, maize, wheat, and barley accounting for over 90% of cereal consumption. The processing of these crops is responsible for the generation of high amounts of by-products which, by virtue of their chemical composition, may be regarded as exceptional raw materials for a circular bioeconomy, in a biorefinery concept [3]. Wheat, in particular, is globally one of the largest cereal crops, used almost exclusively to produce flour, with a yield varying from 73 to 77%. The remaining 23–27% consists primarily of bran, accompanied by a smaller percentage of germ and endosperm. Wheat bran (WB) accounts almost for 25% of the grain weight, and it is largely the most important wheat processing by-product. It has been estimated that nearly 150 million tons of WB are generated per annum [4], and it is therefore considered a raw biomaterial for value-added product recovery, such as polyphenolic antioxidants [5,6]. 

Various health benefits are believed to stem from WB consumption [7], and a number of bioactivities have been attributed to WB phenolics, of which ferulic acid is by far the major constituent [8]. Ferulic acid has long been known for its antioxidant potential [9], and there are several studies pertaining to its implication in battling degenerative diseases, such as cardiovascular disorders and cancer [10]. Owed to these properties, ferulic acid is considered a biomolecule with high potential as a functional food ingredient, but also as an effective food antioxidant and an ideal precursor of bio-vanillin production [11]. However, ferulic acid recovery from WB using conventional extraction methodologies and solvents is particularly low. This is because a large fraction of ferulic acid is bound to chains of arabinoxylans *via* ether or ester linkages [12,13], and therefore it is not readily extractable under conditions regularly employed in a solid-liquid extraction. Alkaline or acid catalysis may greatly improve ferulic acid extraction yield [14,15], yet there is rather a lack of systematic studies on this issue.

The lignocellulosic complex onto which ferulic acid is bound is a matrix with high recalcitrance, requiring relatively harsh conditions for its disintegration. Organosolv treatment is a very promising means in this context, usually as a preliminary step (pretreatment) for the disorganization of the lignocellulosic matrix, and subsequent exploitation of cellulose. It involves exposing the lignocellulosic material to organic solvent/water mixtures at relatively high temperatures for a given resident time, the maxima of which are interdependent [16,17]. The addition of certain catalysts (i.e., sulfuric acid, sodium hydroxide) may greatly increase the efficiency of the process, which involves breaking down ether and ester bonds, such as those that bind ferulic acid onto WB arabinoxylans and lignin. 

In this regard, probably the most challenging prospect in polyphenol recovery from WB is its effective release, with extraction technologies that would ensure both high recovery yield, purity, and stability of the targeted compound, as well as economic feasibility (low cost), with minimal environmental impact. On this conceptual basis, this project was undertaken to establish an organosolv process, aimed at maximizing ferulic acid release/recovery from WB using ethanol, which is a readily available and eco-friendly solvent, and examining the role of acid/alkaline catalysis. Process appraisal was based on both the severity and efficiency, as determined using combined and modified severity factors, and response surface methodology. The assessment of the extracts obtained was based on the determination of ferulic acid content and antioxidant activity. To the best of the authors’ knowledge, such an approach to this issue is reported for the first time. 

## 2. Materials and Methods

### 2.1. Chemicals

Sodium hydroxide and L-ascorbic acid were from Carlo Erba (Milano, Italy). Ferulic acid was purchased from Sigma-Aldrich (Steinheim, Germany). 2,4,6-Tris(2-pyridyl)-s-triazine (TPTZ) was from Fluka (Steinheim, Germany). Sulfuric acid (96%) and sodium carbonate anhydrous were from Penta (Prague, Czech Republic). The stable radical 2,2-diphenyl-1-picrylhydrazyl (DPPH) was from Alfa Aesar (Karlsruhe, Germany). Anhydrous citric acid and iron chloride hexahydrate (FeCl_3_•6H_2_O) were from Merck (Darmstadt, Germany). Folin-Ciocalteu reagent and absolute ethanol were from Panreac (Barcelona, Spain). All solvents used for chromatographic determinations were of appropriate (HPLC) grade.

### 2.2. Wheat Bran

Bran from hard wheat (*Triticum aestivum*), termed WB, was kindly donated by a local mill that regularly processes various cereals to produce several kinds of flours (Katsaris Mills, Karditsa, Greece). The material was shortly transferred to the laboratory, where it was ground in a table coffee mill, and then sieved to yield powder with a mean particle diameter < 300 μm. The ground WB was stored in plastic containers, at 4 °C. 

### 2.3. Alkaline Hydrolysis

The amount of ferulic acid liberated by saponification is generally considered as being the total extractable content of ferulic acid in a plant matrix, and therefore alkaline hydrolysis can be used as a reference procedure [18]. For the alkaline hydrolysis of WB in this study, a previously described procedure was used [15], with slight modifications. Briefly, an exact amount of 1 g of WB was mixed with 75 mL of a 2 M NaOH solution, and stirred for 4 h, at room temperature. The mixture was flushed with nitrogen for 5 min prior to treatment, to eliminate oxygen. After hydrolysis, the mixture was brought to pH 2 using 6 M HCl and centrifuged at 5000× *g*. The transparent supernatant was separated from cell debris and extracted six times with 80 mL ethyl acetate/diethyl ether (1/1). The extracts were combined, and solvent evaporation was accomplished with a rotary evaporator, at 40 °C. The residue was reconstituted in 2 mL 70% (*v*/*v*) aqueous methanol. 

### 2.4. Organosolv Treatment

For all treatments, a volume of 10 mL of solvent was used, and 1 g of WB, to give a liquid-to-solid ratio of 10 mL g^−1^. Initially, the solvent used was ethanol/water mixtures with proportions of 20, 40, 60, and 80% (*v*/*v*). Upon selection of the most appropriate system, sulfuric acid or sodium hydroxide was incorporated to give concentrations of 0.5, 1.0, and 1.5% (*w*/*v*). Treatments were accomplished with a hotplate (Witeg, Wertheim, Germany) and an oil bath, to provide constant heating at 80 °C and stirring at 500 rpm. The temperature of 80 °C was chosen as a safe upper limit to avoid approaching the solvent boiling point, since the solvent systems used were aqueous ethanol mixtures. Thus, the solvent was placed in a Duran vial of 25 mL heated at 80 °C, and then the solid material was added, and the mixture was treated for a resident time of 60, 180, and 300 min. After completion of each treatment, the mixture was allowed to acquire room temperature and centrifuged at 10,000× *g* for 10 min. 

### 2.5. Response Surface Methodology—Process Optimization

The organosolv treatment was optimized by considering the catalyst concentration, *C* (sulfuric acid or sodium hydroxide) and resident time, *t*, as the process (independent) variables, and total polyphenol yield as the response. Since a major part of the free and bound phenolics in WB is represented by ferulic acid [18], the total polyphenol yield would provide a frank account of ferulic acid release. A central composite design was deployed with 11 design points, including three central ones. Codification of the process (independent) variables (*C*, *t*) was in three levels, −1, 0, and 1, and it was accomplished as reported in detail elsewhere [19]. Table 1 presents the levels of the variables both in actual and codified form. The ranges for each variable were chosen based on preliminary experiments, but also pertinent literature data [20,21,22]. Both lack-of-fit and analysis of variance (ANOVA) tests were used to evaluate model statistical significance (R^2^, *p*), as well as the significance of the individual coefficients of each model, considering a minimum level of 95%. 

### 2.6. Process Severity

To compare different treatment conditions, the severity of an organosolv process may be measured by considering the temperature and the resident time, as follows [23,24]:

(1) $$R_{o}=t\;\times \;e^{\left(\frac{T-100}{14.75}\right)}$$

SF = *logR*_o_ (2)

SF represents the severity factor, and *R*_o_ and the value 100 °C are the severity of the reference temperature, respectively. The value 14.75 is an empirical parameter linked to temperature and activation energy. The combined severity factor (CSF) may be regarded as an extended form of SF, and takes into consideration the pH of the treatment medium (solvent), which may also affect biomatrix (WB) disintegration [25]:

(3) $$R_{o}\prime =10^{-\mathrm{pH}}\;\times \;t\;\times \;e^{\left(\frac{T-100}{14.75}\right)}$$

CSF = *logR*_o_′ − pH (4)

However, an alternative approach has also been proposed, which was claimed to provide a fairer comparison of the treatment severities, at widely different treatment pH [25]:

CSF′ = *logR*_o_ + |pH − 7| (5)

Finally, an equation termed modified severity (*M*_o_), that considers catalyst (sulfuric acid or sodium hydroxide) concentration, *C* (% *w*/*v*), has also been proposed [26]:

(6) $$M_{o}=t\times C^{n}\;\times \;e^{\left(\frac{T-100}{14.75}\right)}$$

MSF = *logM*_o_ (7)

The term *n* represents an empirical factor, which has been determined to correspond to 0.849 and 3.90, for treatments carried out with sulfuric acid and sodium hydroxide, respectively [27]. 

### 2.7. High-Performance Liquid Chromatography/Diode Array

A Shimadzu CBM-20A liquid chromatograph (Shimadzu Europa GmbH, Duisburg, Germany) was used, bearing a CTO-20AC column oven, and a Shimadzu SPD-M20A detector. The system was controlled by Shimadzu LC solution software. Chromatographic separations were achieved with a Phenomenex Luna C18(2) column (100 Å, 5 μm, 4.6 × 250 mm) (Phenomenex, Inc., Torrance, CA, USA), kept at a constant temperature of 40 °C. Chromatogram development was performed by using (A) 0.5% aqueous formic acid and (B) 0.5% formic acid in acetonitrile/water (6:4) as eluents, delivered at a flow rate was 1 mL min^−1^. Sample introduction (injection) was made with a 20-μL loop. Details regarding the elution program implemented were given elsewhere [28]. Ferulic acid quantification was accomplished at 320 nm with an external standard, using a ferulic acid calibration curve, with concentrations varying from 0 to 50 μg mL^−1^ (R^2^ = 0.9980). 

### 2.8. Determination of Total Polyphenols, Antiradical Activity, and Ferric-Reducing Power

To determine total polyphenol concentration, a well-established Folin-Ciocalteu protocol was employed [29]. The calibration curve was built with ferulic acid as standard (10–160 mg L^−1^, R^2^ = 0.9984), and the results were expressed as ferulic acid equivalents (FAE). Antiradical activity (A_AR_) measurement was performed with the stable radical probe DPPH, by adopting a previously reported methodology [28]. Results were given as μmol DPPH per g of dry mass (DM). Similarly, the ferric-reducing power assay was executed as previously described [28], and results were given as μmol ascorbic acid equivalents (AAE) per g of dry mass (DM). For both A_AR_ and P_R_ assays, samples were diluted 1/20 with 1 M formic acid in methanol prior to determinations.

### 2.9. Statistics

At least two repetitions were performed for all organosolv treatments, while the analytical determinations were repeated at least in triplicate. All values were given as average (±standard deviation). Linear regressions were carried out with SigmaPlot™ 12.5 (Systat Software Inc., San Jose, CA, USA), at a significance level of at least 95% (*p* < 0.05). The experimental design of the response surface methodology and the relevant statistical processing (lack-of-fit, ANOVA) were carried out with JMP™ Pro 13.

## 3. Results and Discussion

### 3.1. Selection of Ethanol/Water Proportion

**Figure 1:**
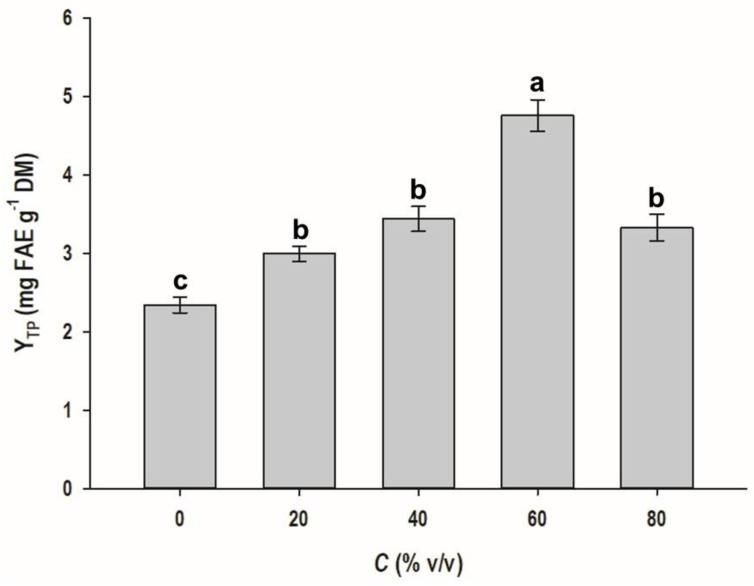
Effect of water/ethanol proportion on the extraction yield in polyphenols from wheat bran. Extractions were accomplished with the liquid-to-solid ratio of 10 mL g^-1^, at 80 °C, for 180 min. Columns assigned with a, b, and c represent values with statistically significant difference (*p* < 0.05).

As a first step in the development of an effective process for polyphenol recovery from WB, the proportion of ethanol/water was tested, to ascertain the optimal ethanol concentration. Thus, a range of hydroethanolic solvents was used, with ethanol concentration varying from 0 (pure water) to 80% (*v*/*v*). In Figure 1 can be seen that up to 60% ethanol, there was a gradual increase in total polyphenol extraction yield (Y_TP_). By contrast, increasing ethanol concentration from 60 to 80% resulted in a significant drop in Y_TP_ (*p* < 0.05). Therefore, the hydroethanolic solvent with 60% ethanol was chosen as the most appropriate, and it was used for any further treatment. 

### 3.2. Process Severity—Correlation with Extraction Yield

The severity of a process reflects the harshness of the conditions employed, and this can be estimated by determining the severity factor. In an organosolv process performed in the presence of an acid or alkali catalyst, pH is an integral part of the severity [25]. Therefore, the combined severity factor that takes into account the pH of the solvent used is more descriptive, in that it also considers the influence of the solvent acidity or alkalinity. Furthermore, as catalyst concentration may also significantly affect lignocellulosic biomass untangling [27], such an effect should also be examined with regard to polyphenol release from WB. At this point, it should be emphasized that polyphenol release from WB may be favored under both acidic and alkaline conditions [30].

**Figure 2:**
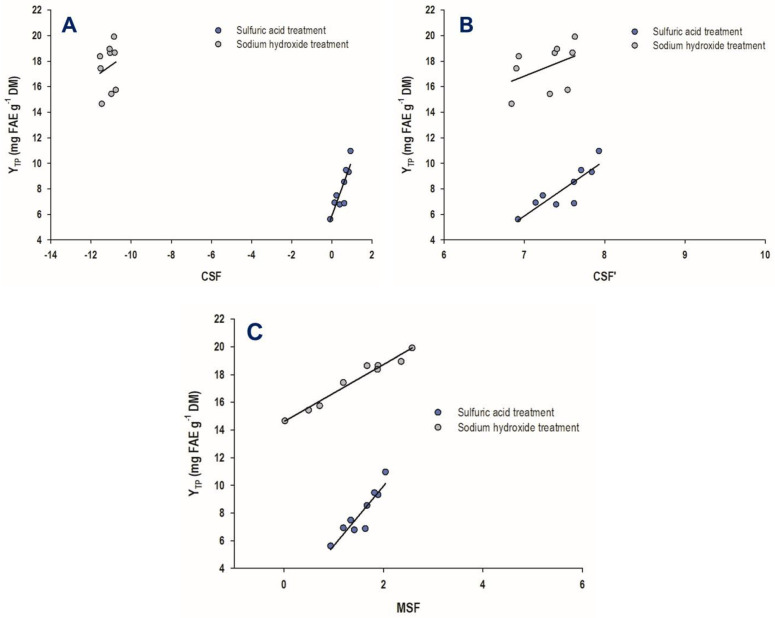
Linear regressions established between the yield in total polyphenols (Y_TP_) and the combined severity factor (**A**), alternative combined severity factor (**B**), and modified severity factor (**C**).

On this basis, the first approach attempted was the determination of the combined severity factor (CSF), using Equation (4). The CSF values for the treatment with sulfuric acid varied from −0.08 to 0.93, whereas those for the treatment with sodium hydroxide varied from −11.46 to −10.85 (Table 2). This outcome indicated high differences in the severity of the process, as given by the CSF. When CSF values were plotted against Y_TP_ (Figure 2A), the following linear models were derived:

Y_TP(SuAc)_ = 4.34CSF_SuAc_ + 5.88 (R^2^ = 0.76, *p* = 0.0023) (8)

Y_TP(SoHy)_ = 1.10CSF_SoHy_ + 29.79 (R^2^ = 0.04, *p* = 0.6278) (9)

where SuAc and SoHy denote the treatment with sulfuric and sodium hydroxide, respectively.

The strong and significant (*p* < 0.05) correlation found for the treatment with sulfuric acid was clear evidence that polyphenol release was directly proportional to process severity. On the contrary, no such evidence emerged for the treatment with sodium hydroxide. 

When the alternative combined severity factor (CSF′) was considered, using Equation (5), the values obtained exhibited high convergence. More particularly, for the treatment with sulfuric acid, CSF′ values ranged from 6.92 to 7.93, and the corresponding values for the sodium hydroxide treatment were from 6.84 to 7.63 (Table 2). Linear correlations between CSF′ and Y_TP_ (Figure 2B) afforded the models shown below:

Y_TP(SuAc)_ = 4.34CSF′_SuAc_ − 24.50 (R^2^ = 0.76, *p* = 0.0023) (10)

Y_TP(SoHy)_ = 2.50CSF′_SoHy_ − 0.68 (R^2^ = 0.18, *p* = 0.2517) (11)

Similar to what was observed for the correlation of CSF with Y_TP_, Equations (10) and (11) indicated that the severity of the sulfuric acid treatment was strongly correlated with polyphenol release, but the severity of the sodium hydroxide treatment did not display such a trend. 

The determination of the modified severity factor (MSF) using Equation (7) showed that the ranges for the sulfuric acid and sodium hydroxide treatments were from 0.93 to 2.04 and from 0.02 to 2.58, respectively (Table 2). Linear regression between MSF and Y_TP_ (Figure 2C) yielded the following models:

Y_TP(SuAc)_ = 4.31MSF_SuAc_ + 1.33 (R^2^ = 0.83, *p* = 0.0006) (12)

Y_TP(SoHy)_ = 2.06MSF_SoHy_ + 14.61 (R^2^ = 0.96, *p* < 0.0001) (13)

In this case, and specifically for the sodium hydroxide treatment, it was revealed that the models exhibited excellent adjustment to the experimental data. On this ground, it could be argued that using Equation (13), Y_TP_ could be predicted by the MSF with high reliability. Moreover, both Equations (12) and (13) concurred that polyphenol release from WB using either sulfuric acid or sodium hydroxide was strongly linked to catalyst concentration.

On the ground of the models represented by Equations (8), (10), (12), and (13), it could be supported that total polyphenol yield was directly proportional to process severity, in line with total polyphenol extraction from WB with pressurized water/ethanol mixtures [31]. However, irrespective of severity, Y_TP_ was always significantly higher when sodium hydroxide was used as a catalyst. For both processes with sulfuric acid and sodium hydroxide, the highest Y_TP_ was achieved with a catalyst concentration of 1.5% and a resident time of 300 min. Under these conditions, the process with sodium hydroxide afforded 1.82-fold higher Y_TP_ compared to sulfuric acid (Table 2). This finding was in accordance with several previous examinations, where the superiority of sodium hydroxide hydrolysis over acid hydrolysis in releasing polyphenols from WB was demonstrated [15,32,33]. Although it has been proposed that acid hydrolysis may complement the alkali-catalyzed one [30], the abundance of literature data strongly recommended polyphenol liberation under alkaline conditions as the highest-performing process [18]. 

### 3.3. Process Optimization with Response Surface Methodology

**Figure 3:**
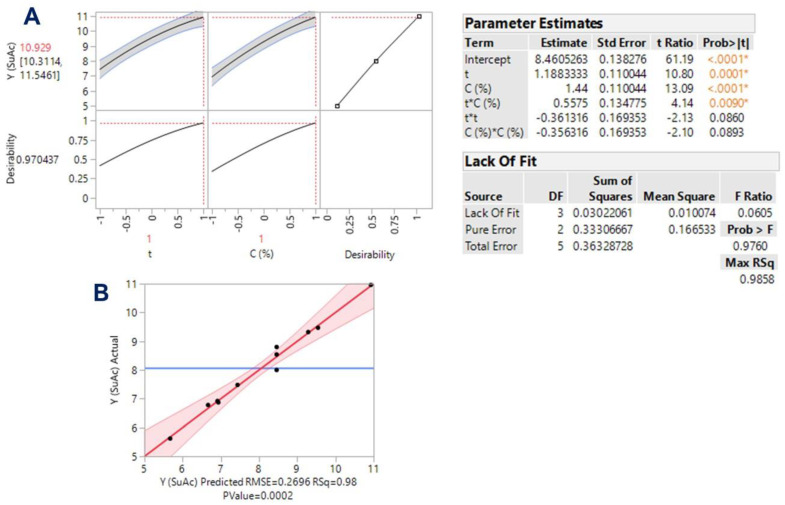
Plot showing the function of desirability (**A**) and the actual vs. predicted values (**B**) of the yield in total polyphenols (Y_TP_), for the optimization of polyphenol release from WB using sulfuric acid (SuAc) as a catalyst. The inset tables present the statistical data related to the model built based on the response surface methodology. Values designated with different colors and asterisks are statistically significant.

**Figure 4:**
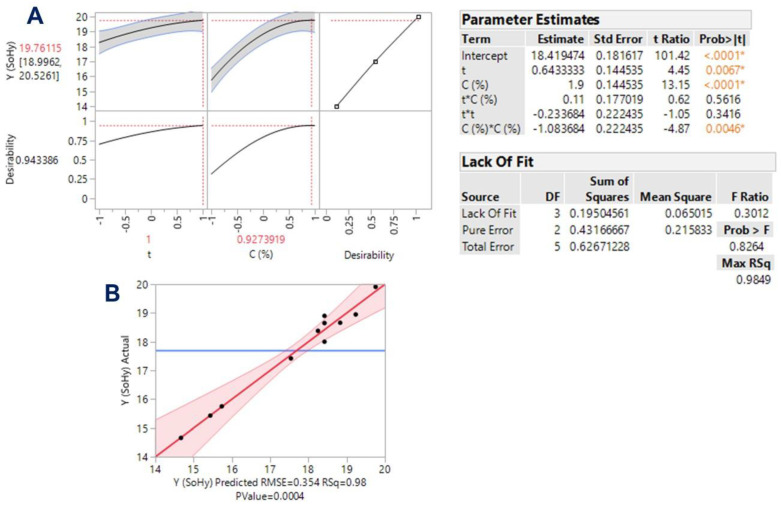
Plot showing the function of desirability (**A**) and the actual vs. predicted values (**B**), for the optimization of polyphenol release from WB using sodium hydroxide (SoHy) as a catalyst. The inset tables present the statistical data related to the model built based on the response surface methodology. Values designated with different colors and asterisks are statistically significant.

The evidence that emerged from the linear models implicating catalyst concentration strongly suggested that this variable may play a prominent role in polyphenol release from WB. Thus, process modeling was also approached through response surface optimization, by considering time, *t*, and catalyst concentration, *C*, as the independent variables. The methodology implemented aimed at appraising the effect of these crucial variables, but also to identify possible synergistic (cross) functions among them. Assessment of the models derived, and response surface optimization was based on statistical elaboration (lack-of-fit and analysis of variance (ANOVA) tests) (Figure 3 and Figure 4), considering the proximity of the predicted and measured values (Table 3). 

The derived mathematical models, containing only the significant equation terms, were as follows:

Y_TP(SuAc)_ = 8.46 + 1.18X_1_ + 1.44X_2_ + 0.56X_1_X_2_ (14)

Y_TP(SoHy)_ = 18.42 + 0.64X_1_ + 1.90X_2_ − 1.08X_2_^2^ (15)

**Figure 5:**
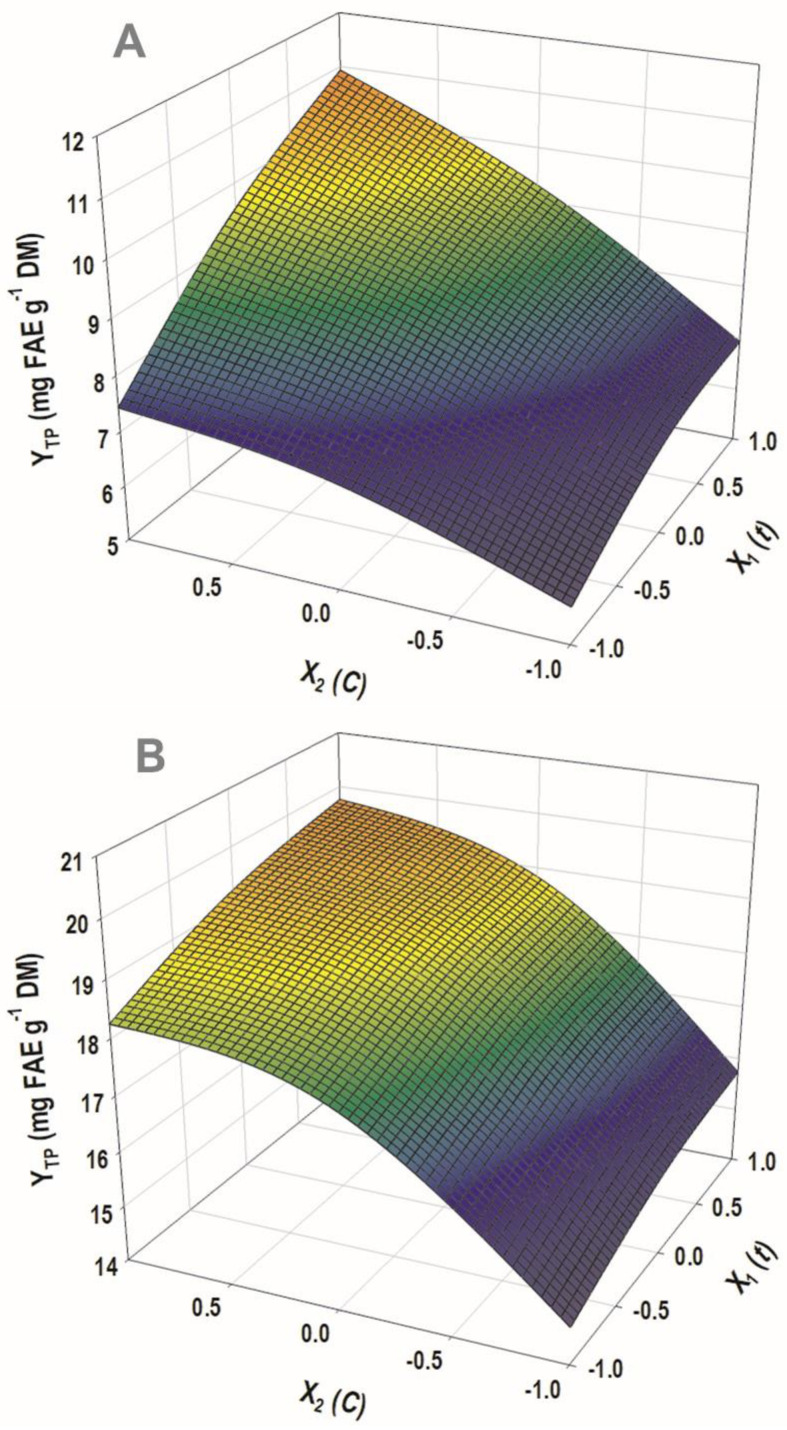
The 3D diagrams representing the effect of the process variables (*C*, *t*) on the yield in total polyphenols (Y_TP_). (**A**), sulfuric acid-catalyzed process; (**B**), sodium hydroxide-catalyzed process.

Both models had R^2^ were equal to 0.98, and, considering a confidence interval of at least 95%, the *p*-value determined for lack-of-fit was highly significant (Figure 3B and Figure 4B). On this basis, it could be argued that both models showed outstanding fitting to the experimental values. The three-dimensional plots constructed using the derived models portrayed the effect of the process variables on the response (Y_TP_) and depicted the differences between the two catalysts tested (Figure 5). 

For the process performed with sulfuric acid, both *t* (X_1_) and *C* (X_2_) were highly significant (*p* < 0.0001), as was the cross term between them (X_1_X_2_) (Figure 3, inset table “Parameter Estimates”). On the other hand, the quadratic effects of either *t* or *C* were non-significant. Likewise, both X_1_ and X_2_ exerted a significant effect on Y_TP_ when the process was carried out with sodium hydroxide as a catalyst (Figure 4). However, in this case, no significant cross-terms were detected, but X_2_ exhibited a quadratic effect (X_2_^2^). This effect was visually manifested by the curvature displayed in the response surface (Figure 5B). The positive terms for both *t* and *C*, in the case of sulfuric acid catalysis, suggested that further increases in both variables would lead to higher Y_TP_. On the contrary, the negative quadratic term in the case of sodium hydroxide catalysis showed that beyond a certain limit, no benefit could be gained by increasing catalyst concentration. 

**Figure 6:**
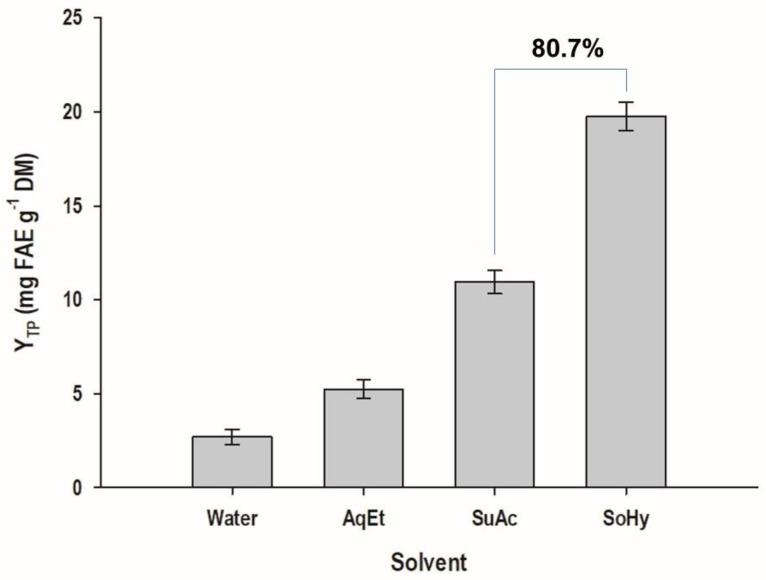
Yield in total polyphenols (Y_TP_) achieved after implementing sulfuric acid- and sodium hydroxide-catalyzed treatments of wheat bran, under optimized conditions. Water and aqueous ethanol (AqEt) are control treatments. SuAc and SoHy denote treatments catalyzed by sulfuric acid and sodium hydroxide, respectively.

Indeed, the desirability function enabled the determination of the optimal values for both *t* and *C*, as well as the maximum predicted response for each process tested (Figure 3A and Figure 4A). It can be seen that in the case of sodium hydroxide catalysis, X_2_ reached an inflection point at 0.927, which corresponded to a concentration of 1.46%. Under optimized conditions, the highest Y_TP_ estimated for the process with sulfuric and sodium hydroxide were 10.93 ± 0.62 and 19.76 ± 0.76 mg FAE g^−1^ DM, respectively. These values matched exactly those determined experimentally, when treatments were carried out with 1.5% catalyst concentration, for 300 min (Table 2). If the optimal values (*t* = 300 min, *C* = 1.5% for sulfuric acid; *t* = 300 min, *C* = 1.46% for sodium hydroxide) were incorporated into Equation (7), the corresponding MSF would be 2.04 and 2.53. Thus, it could be argued that, in spite of a small difference in severity, sodium hydroxide largely outperformed sulfuric acid catalysis, providing almost 81% higher Y_TP_ (Figure 6).

The level of 19.76 ± 0.76 mg FAE g^−1^ DM achieved with sodium hydroxide catalysis was exceptionally high, considering that other investigations reported Y_TP_ values for aqueous ethanol extraction ranging from 0.84 [34] to 3.05 mg FAE g^−1^ DM [35]. Higher levels of 4.66 mg gallic acid equivalents g^−1^ DM were achieved with 50% acetone [36], and 5.90 mg caffeic acid equivalents g^−1^ DM with a lactic acid/choline chloride deep eutectic solvent [35]. By employing alkaline hydrolysis, other authors reported levels of 12.20 mg FAE g^−1^ DM, [37], and levels ranging from 2.30–5.39 mg FAE g^−1^ DM [38], but the steam explosion was shown to be even more efficient, yielding 27.71 mg GAE g^−1^ DM [39]. However, yields as high as 94.62 mg FAE g^−1^ DM have been achieved using a ternary deep eutectic solvent, composed of glycerol/citric acid/glycine and ultrasonication pretreatment [33]. 

### 3.4. Polyphenolic Composition

**Figure 7:**
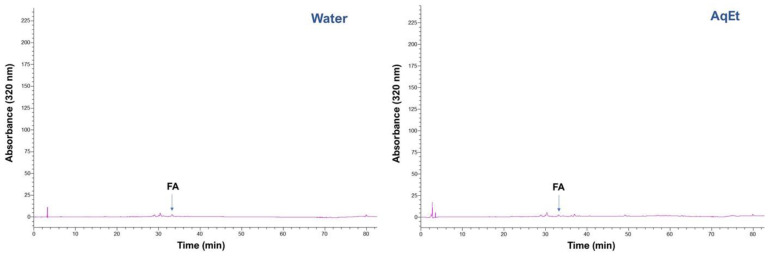

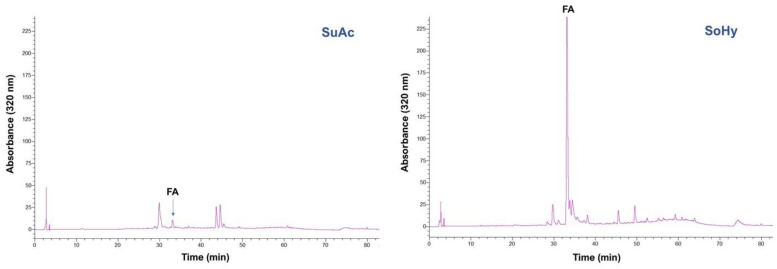
Representative HPLC traces recorded at 320 nm, of all the extracts tested. Water and aqueous ethanol (AqEt) extracts were used as control samples. SuAc and SoHy denote the extracts obtained with the sulfuric acid and sodium hydroxide-catalyzed process, respectively, under the conditions optimized through the response surface methodology. FA denotes ferulic acid.

The polyphenolic profile of the extract obtained with 1.5% sodium hydroxide was compared to that obtained with 1.5% sulfuric acid, to trace compositional differences that might have been raised as a response to the different catalysts used. Furthermore, the composition of control extracts produced with water and 60% aqueous ethanol was also considered, to better illustrate the effect of catalysts. In Figure 7 can be seen that, compared to all other treatments, the sodium hydroxide catalysis had a striking effect, proving an extract highly enriched in ferulic acid. 

Generally, the polyphenolic profile of WB extracts is largely dominated by ferulic acid, accompanied by lower amounts of *p*-coumaric acid, and a much lower proportion of other constituents, including syringic and vanillic acids [14,40]. However, in the extracts analyzed, no *p*-coumaric acid was detected irrespective of the catalyst used. The quantitative analysis showed that the amount of ferulic acid recovered was 1945 μg g^−1^ DM, which was almost 4.5% higher than the amount recovered by the control alkaline hydrolysis (Table 4). By consenting that the recovery achieved with alkaline hydrolysis represented roughly the total ferulic acid content in WB, then, under the optimized conditions employed, the sodium hydroxide-catalyzed process recovered the maximum amount of ferulic acid. By contrast, the sulfuric acid-catalyzed process afforded only 75.93 μg of ferulic acid g^−1^ DM, which represented approximately 4.1% of the total ferulic acid content. This finding pointed emphatically to the exceptionally high efficiency of the sodium hydroxide-catalyzed process in releasing ferulic acid from WB. 

In wheat tissues, including straw and bran, ferulic acid is bound to arabinoxylan constituents of cell walls through ester bonds [12,41]. Ester bonds are prone to alkaline hydrolysis, hence the increased ferulic acid release found. However, in matrices such as wheat straw, hydroxycinnamates primarily represented by ferulic acid may also act as cross-linking substances between polysaccharides and lignins. In this case, too, ferulic acid is mainly bound to polysaccharides with alkali-labile esters bonds [42]. Moreover, ferulic acid is also a constituent of the lignin network, connected with ester bonds. Therefore, lignin solubilization could further enhance ferulic acid release [43]. In this case, the role of ethanol may be crucial, assisting lignin solubilization [44], but also facilitating the accessibility of ester bonds by sodium hydroxide, which is a rate-limiting step in the hydrolysis process [43]. Ether-linked ferulic acid may be cleaved only upon acid catalysis, but such a process requires temperatures well above 80 °C used in this study. With acid-catalyzed treatment, hemicellulose fractions may be solubilized, but the ester-linked ferulic acid remains unaffected. 

On the other hand, ester bonds may be broken down even under mild alkaline conditions, and acceleration of the process may be achieved by appropriately regulating temperature [43]. Nevertheless, treatments at 140 °C for over 40 min were claimed to be unsuitable, leading probably to ferulic acid degradation [45]. Yet, pressurized hot water treatment was demonstrated to yield maximum ferulic acid amount at 200 °C, with only 3.5 min resident time [46], but treatment with pressurized water/ethanol mixtures required 74 min at 160 °C, to release maximum ferulic acid amount [31]. Considering that in thermal processes time and temperature are interdependent variables, adjustment of treatment temperature and/or time to optimum levels would be crucial to maximizing ferulic acid recovery. 

Furthermore, lignin breakdown and solubilization may further enhance ferulic acid release. It has been observed that, during ethanol organosolv treatment, cleavage of *α*-aryl and *β*-aryl ether linkages largely contribute to lignin breakdown [42,44]. These reactions split large lignin polymers into smaller fractions, enabling higher lignin solubilization. Such reactions may take place either in the presence of an acid or alkali catalyst. However, alkaline treatments may greatly facilitate lignin solubilization [47], which may be further enhanced by the presence of ethanol [16]. Ferulic acid release has been tightly associated with alkali-solubilized lignin [43], and this could be another reason for its extended release under the condition employed in this study. 

The amount of ferulic acid recovered by the alkali-catalyzed process (1945.76 μg g^−1^ DM) was close to levels of 1918 and 2193 μg g^−1^ DM previously reported [14,30], and significantly higher than 1660.60 μg g^−1^ DM [15], but far greater than that found in other studies, such as 51.93 [48], 226.8 [31], 231.00 [34], and 406.14 μg g^−1^ DM [49]. However, higher yields of 2391.56 [39], 3084.20 [40], and 3910 μg g^−1^ DM [50] have also been achieved. 

### 3.5. Antioxidant Characteristics of the Extracts

**Figure 8:**
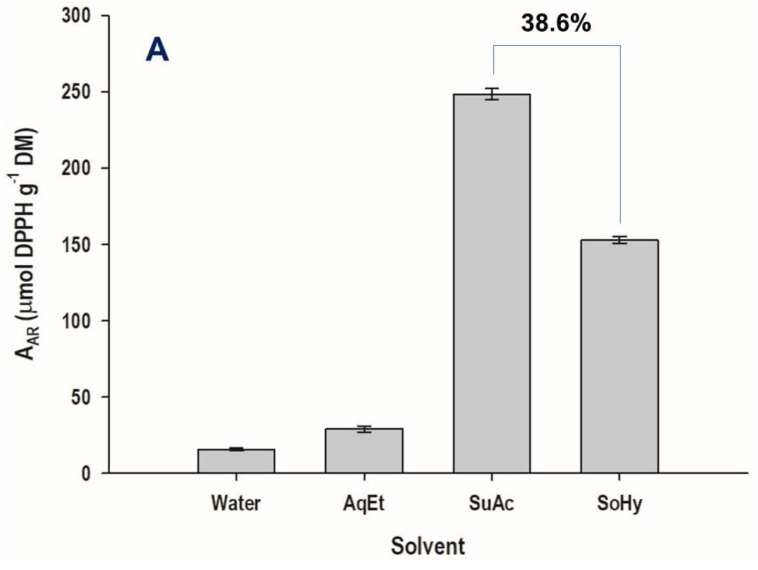

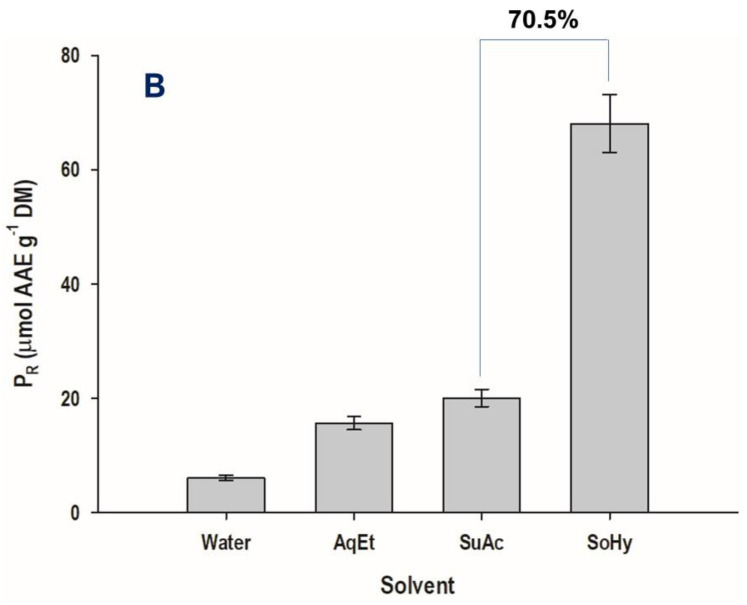
Antiradical activity (A_AR_) (**A**) and ferric-reducing power (P_R_) (**B**) of the extracts produced with sulfuric acid- and sodium hydroxide-catalyzed treatments of wheat bran, under optimized conditions. Water and aqueous ethanol (AqEt) are control treatments. SuAc and SoHy denote treatments catalyzed by sulfuric acid and sodium hydroxide, respectively.

The measurement of the antioxidant properties of the extracts produced with either acid or alkali catalysis gave contradictory outcomes. Thus, while the extract produced with acid catalysis displayed almost 39% higher antiradical activity (A_AR_) compared to the extract generated with alkaline catalysis, the latter exhibited 70% higher ferric-reducing power (P_R_) compared to the former (Figure 8). 

It is irrefutable that, in WB extracts, ferulic acid plays a prominent role in the expression of antioxidant activity and may be the major antioxidant contributor [15,51]. This option was also corroborated by hydrolysis studies, where ferulic acid-enriched extracts obtained after alkaline hydrolysis possessed more powerful antiradical activity than the extracts obtained with acid hydrolysis [14]. 

Aside from that, the overall antiradical activity of WB extracts has been significantly correlated with the total polyphenol concentration [38,52,53]. On this ground, the results on A_AR_ would appear rather a paradox, given the vast difference in ferulic acid but also total polyphenol content between the acid and the alkali-produced extracts. By contrast, results concerning P_R_ were consistent in this regard. A similar phenomenon has been previously seen, where the A_AR_ of WB polyphenol-containing extracts produced with various solvents did not coincide with P_R_ [33,35]. Therefore, it could be argued that other compounds in the extracts that resulted from acid catalysis could affect the overall antioxidant behavior of WB extracts. Additionally, it should be pointed out that combinations of individual polyphenols may result in synergistic and/or antagonistic effects, as observed for various polyphenol mixtures [54,55], and the final antiradical activity or ferric-reducing power determined may reflect the integration of such phenomena.

## 4. Conclusions

In this study, a process for polyphenol release from wheat bran was modeled, using severity but also response surface methodology. The use of hydroethanolic mixtures and either sulfuric acid or sodium hydroxide as catalysts revealed that, under comparable combined severity, the alkali-catalyzed process is exceptionally more efficient than the acid-catalyzed one. At constant temperature, polyphenol release efficiency was significantly affected by both resident time and catalyst concentration. Furthermore, it was found that the alkali-catalyzed process may afford ferulic acid yields several times higher than the acid-catalyzed one. Yet, discrepancies were observed concerning the antioxidant properties of the extracts obtained, suggesting that constituents other than ferulic acid could be implicated. The final outcome would dictate that ethanol organosolv treatment of wheat bran, assisted by mild alkaline catalysis, could be a very effective means of releasing and recovering pure ferulic acid, which may be used in the food, pharmaceutical, and cosmetics industry as a functional ingredient. This process could be integrated into wider strategies of biorefinery, thus contributing to establishing sustainable routes of wheat by-product exploitation, in a circular economy context. 

## Figures and Tables

**Table 1:** The levels (actual and coded) of the process variables used to set up the experimental design.

Process (Independent) Variables	Codes	Coded Variable Level		
		−1	0	1
*t* (min)	X_1_	60	180	300
*C* (%, *w*/*v*)	X_2_	0.5	1.0	1.5

**Table 2:** Combined severity factor (CSF), alternative combined severity factor (CSF′), and modified severity factor (MSF) values, along with the corresponding total polyphenol yield (Y_TP_) values, for the various treatments tested. Assignments SuAc and SoHy correspond to treatments with sulfuric acid and sodium hydroxide.

*C* (% *w*/*v*)	*t* (min)	CSF		CSF′		MSF		Y_TP_ (mg FAE g^−1^ DM)	
		SuAc	SoHy	SuAc	SoHy	SuAc	SoHy	SuAc	SoHy
0.5	60	−0.08	−11.46	6.92	6.84	0.93	0.02	5.62	14.66
	180	0.40	−10.98	7.40	7.32	1.41	0.49	6.78	15.43
	300	0.62	−10.76	7.62	7.54	1.63	0.71	6.87	15.75
1.0	60	0.14	−11.52	7.14	6.90	1.19	1.19	6.92	17.42
	180	0.62	−11.04	7.62	7.38	1.67	1.67	8.54	18.65
	300	0.84	−10.82	7.84	7.60	1.89	1.89	9.32	18.66
1.5	60	0.23	−11.55	7.23	6.93	1.34	1.88	7.48	18.38
	180	0.71	−11.07	7.71	7.41	1.82	2.35	9.47	18.95
	300	0.93	−10.85	7.93	7.63	2.04	2.58	10.96	19.91

**Table 3:** The design points included in the response surface methodology, and the values (measured and predicted) of total polyphenol yield (Y_TP_), obtained for both acid- and alkali-catalyzed treatments.

Design Point	Independent Variables		Response (Y_TP_, mg FAE g^−1^ DM)			
	X_1_ (*t*)	X_2_ (*C*)	SuAc		SoHy	
			Measured	Predicted	Measured	Predicted
1	−1	−1	5.62	5.67	14.66	14.67
2	−1	1	7.48	7.44	18.38	18.25
3	1	−1	6.87	6.93	15.75	15.74
4	1	1	10.96	10.93	19.91	19.76
5	−1	0	6.92	6.91	17.42	17.54
6	1	0	9.32	9.29	18.66	18.83
7	0	−1	6.78	6.66	15.43	15.44
8	0	1	9.47	9.54	18.95	19.24
9	0	0	8.54	8.46	18.65	18.42
10	0	0	8.00	8.46	18.00	18.42
11	0	0	8.80	8.46	18.90	18.42

**Table 4:** Ferulic acid content of wheat bran determined after performing various treatments. Values represent means of triplicate treatments ± standard deviation.

Solvent	FA (μg g^−1^ DM)
Hydrolysate	1861.29 ± 20.40 ^a^
Water	22.52 ± 1.52 ^b^
60% Ethanol	21.96 ± 1.98 ^b^
60% Ethanol—1.5% Sulfuric acid	75.93 ± 5.44 ^b^
60% Ethanol—1.5% Sodium hydroxide	1945.76 ± 32.04 ^a^

^a, b^ Different letters denote statistically different values.

## Data Availability

Data are contained within the article.
